# Transforming Growth Factor-β, Macrophage Colony-Stimulating Factor and C-Reactive Protein Levels Correlate with CD14^high^CD16^+^ Monocyte Induction and Activation in Trauma Patients

**DOI:** 10.1371/journal.pone.0052406

**Published:** 2012-12-28

**Authors:** Sonlee D. West, Daniel Goldberg, Anna Ziegler, Michael Krencicki, Terry W. Du Clos, Carolyn Mold

**Affiliations:** 1 Department of Surgery, University of New Mexico Health Sciences Center, Albuquerque, New Mexico, United States of America; 2 Department of Molecular Genetics and Microbiology, University of New Mexico Health Sciences Center, Albuquerque, New Mexico, United States of America; 3 Department of Internal Medicine, University of New Mexico Health Sciences Center, Albuquerque, New Mexico, United States of America; 4 VA Medical Center, University of New Mexico Health Sciences Center, Albuquerque, New Mexico, United States of America; University of Cincinnati, United States of America

## Abstract

Severe injury remains a leading cause of death and morbidity in patients under 40, with the number of annual trauma-related deaths approaching 160,000 in the United States. Patients who survive the initial trauma and post-traumatic resuscitation are at risk for immune dysregulation, which contributes to late mortality and accounts for approximately 20% of deaths after traumatic injury. This post-traumatic immunosuppressed state has been attributed to over-expression of anti-inflammatory mediators in an effort to restore host homeostasis. We measured a panel of monocyte markers and cytokines in 50 severely injured trauma patients for 3 days following admission. We made the novel observation that the subpopulation of monocytes expressing high levels of CD14 and CD16 was expanded in the majority of patients. These cells also expressed CD163 consistent with differentiation into alternatively activated macrophages with potential regulatory or wound-healing activity. We examined factors in trauma plasma that may contribute to the generation and activation of these cells. The percentage of CD14^high^CD16^+^ monocytes after trauma correlated strongly with plasma C-reactive protein (CRP) transforming growth factor-β (TGF-β), and macrophage colony-stimulating factor (M-CSF) levels. We demonstrate a role for TGF-β and M-CSF, but not CRP in generating these cells using monocytes from healthy volunteers incubated with plasma from trauma patients. CD16 is a receptor for CRP and IgG, and we showed that monocytes differentiated to the CD14^high^CD16^+^ phenotype produced anti-inflammatory cytokines in response to acute phase concentrations of CRP. The role of these cells in immunosuppression following trauma is an area of ongoing investigation.

## Introduction

Severe traumatic injury is a leading cause of death and morbidity in patients under 40. In patients who survive the initial trauma and post-traumatic resuscitation, innate immunity induces both local and systemic release of pro-inflammatory cytokines, acute phase proteins, hormones and other inflammatory mediators. The excessive release of these mediators plays an important role in the pathogenesis of shock [1], [2]. In parallel to this pro-inflammatory response, there is an anti-inflammatory response characterized by the release of anti-inflammatory cytokines and mediators [3], [4] that helps restore immune equilibrium. This compensatory anti-inflammatory response may be deleterious by dampening the immune system to the extent that the immune response is compromised and the patient becomes susceptible to infection [5], [6], [7].

Monocytes and macrophages are key initiators and regulators of the innate immune response in trauma, shock and sepsis. Subpopulations of monocytes have distinct and specific roles in the spectrum of the immune response that include, but are not limited to, cytokine production and antigen presentation [8], [9]. Monocytes can be identified as belonging to 1 of 3 subpopulations by their surface marker expression, function and cytokine production [8]. Approximately 90% of monocytes in healthy individuals belong to the classic subpopulation that expresses a high level of CD14, a co-receptor for LPS, without expressing FcγRIIIa (CD16), a receptor for IgG and C-reactive protein (CRP). Two minor subpopulations, termed “intermediate” and “non-classical” express CD16 with high or low CD14 expression, respectively. The CD16+ subpopulations, which are normally between 5–10% of circulating monocytes [8], have been shown to expand during certain inflammatory illnesses, but little is known of the role of the expansion of these subpopulations in the pathogenesis of disease.

An additional population of “deactivated monocytes” has been described following trauma and associated with sepsis. These monocytes are CD14^+^CD16^−^. They fail to make TNF-α when stimulated with bacterial lipopolysaccharide (LPS) and have decreased expression of HLA class II molecules. More than 80% of peripheral blood mononuclear cells (PBMC) in healthy individuals are HLA-DR+ [10].

CD14^low^CD16^+^ “non-classical” monocytes have been well characterized and are known to expand during infection and inflammation [11], [12], [13]. These monocytes are generally regarded as proinflammatory because they produce more TNF-α than the classic subpopulation with LPS stimulation and produce little to no IL-10.

The more recently described CD14^high^CD16^+^ “intermediate” subpopulation was found to increase in parallel to the CD14^low^CD16^+^ monocytes in septic newborns [14]. This subpopulation of monocytes has been associated with an increased expression of anti-inflammatory mediators. These monocytes are the main producers of the anti-inflammatory cytokine IL-10 in response to LPS stimulation [11]. In addition these monocytes and “classical” monocytes when stimulated by alternative activation pathways express the CD163 hemoglobin scavenger receptor [15]. CD163 is responsible for clearance of hemoglobin-haptoglobin (Hb-Hp) complexes, mediating endocytosis of the complex, release of IL-10 and expression of HO-1 [11], [16], [17]. The heme subunit is then degraded by the rate-limiting enzyme HO-1 yielding biliverdin, free iron and carbon monoxide, all of which have strong anti-inflammatory effects [18], [19]. HO-1 and IL-10 in turn upregulate CD163 expression in a positive feedback loop [16],[17]. The CD14^high^CD16^+^ monocyte subpopulation has not been studied in trauma patients.

We hypothesize that expansion of the CD14^high^CD16^+^ subpopulation of monocytes after trauma is part of the anti-inflammatory response, resulting in an increase in the production of anti-inflammatory mediators, including IL-10 and IL-1RA. The current study examines factors in trauma plasma that induce CD16, CD163 and HO-1 expression by healthy monocytes and activate CD14^high^CD16^+^ monocytes to produce cytokines. Additional studies are needed to directly demonstrate the functional activity of the CD14^high^CD16^+^ subpopulation of monocytes following trauma.

## Patients and Methods

### Patients and Ethics Statement

We collected blood from 50 severely injured trauma patients, admitted to the Trauma Service at the University of New Mexico Hospital. All samples were obtained in accordance with guidelines and under protocols approved by the Human Research Review Committee at the University of New Mexico Health Sciences Center. Enrollment criteria included ICU admission or prospective Injury Severity Score of greater than 16, as a marker of severe injury, age greater than 18 years, and a negative pregnancy test. Of the patients, 35 were men and 15 were women, with a mean average age of 39.6 (range 18–73). The mean Injury Severity Score was 30 (range 9–55). The majority of patients (86%) suffered blunt trauma and multisystem injuries (90%). No patient was treated with any immunosuppressant or corticosteroid. No patients were included that were admitted to the ICU for merely observation status. Results from a portion of this patient cohort were previously published [20]. These patients were a prospectively collected convenience sample of sequential patients who had suffered severe trauma and were able to provide written informed consent or had a legally authorized representative who was able to provide written informed consent. Human peripheral blood monocytes from healthy donors were collected from participants who provided written informed consent. During this study, 26 of 50 patients developed infections, 6 developed multiple organ dysfunction and two patients died.

### Blood Samples

We collected blood samples from the patients at enrollment (within 24 hours of admission) and at 48 and 72 hours. A panel of monocyte markers was determined at 48 hours. Plasma cytokine concentrations and CRP levels were determined from the 24, 48 and 72-hour blood samples. Controls were healthy volunteers and included 3 males and 4 females with an average age of 38, which is representative of the overall patient population. No age or gender matching was performed between controls and patients.

### Plasma Cytokine Concentrations

Blood was collected in heparinized tubes from patients at 24, 48 and 72 hours. Samples were centrifuged at 1000 rpm for 10 minutes and then plasma was collected and stored at -80°C for cytokine concentrations and CRP levels. Plasma was analyzed for 10 cytokines (TNF-α, IL-6, IL-8, IL-10, IL-12, IL-1RA, IL-1β, IFN-γ, MCP-1, MIP-1α) using a Milliplex Human Cytokine Immunoassay according to manufacturer’s instructions (Millipore). CRP, M-CSF and TGF-β concentrations were determined with enzyme-linked immunosorbent assay (ELISA) according to manufacturer’s instructions (BD Bioscience).

### Monocyte Surface Marker Expression

Expression of CD14, CD16, CD163 and HLA-DR on patient monocytes was determined by flow cytometry. Whole blood samples were collected in EDTA-treated tubes at 48 hours, and 200 µl was used for measurements. The following mAb were used: FITC anti-CD14 (Miltenyi Biotec) PerCP-Cy5.5 anti-HLA-DR (BD Bioscience), phycoerythrin (PE) and PerCP-Cy5.5 anti-CD16 (BD Bioscience) and PE anti-CD163 (BD Bioscience) and corresponding isotype controls. Cells were incubated with the mAb for 10 minutes in the dark. RBC were lysed with 2 ml of RBC lysis solution (eBioscience) at room temperature for 10 minutes. Cells were washed once with phosphate buffered saline (PBS) and twice with 3 ml of PAB (0.1% BSA/0.05% sodium azide in PBS) staining buffer. Cells were then fixed with 2% paraformaldehyde and the fluorescence of each sample was analyzed with a flow cytometer (BD Bioscience) and FlowJo Software (Tree Star, Inc). Results are expressed as the geometric mean fluorescence intensity (GMFI) after subtracting the GMFI for the corresponding isotype control (ΔGMFI).

### Determination of Intracellular HO-1

Intracellular HO-1 in patient monocytes was measured by flow cytometry. Whole blood samples were collected at 48 hours in EDTA-treated tubes, and 200 µl was used for measurements. Twenty microliters of PE anti-CD14 (BD Biosciences) was added and the samples were incubated for 10 minutes at room temperature. RBC were lysed with RBC lysis solution (eBioscience) at room temperature for 10 minutes. Cells were washed once with PBS and twice with PAB. One milliliter of 3% paraformaldehyde (Sigma) was used to fix the cells at room temperature for 10 minutes. After centrifugation, samples were washed with PBS, and permeabilized with 1 ml of 0.2% saponin/0.1% BSA/0.05% azide in PBS for 30 minutes. Samples were then washed in the permeabilization buffer (0.2% saponin/0.1% BSA/0.05% azide in PBS). After centrifugation, 2 µl of rabbit anti-human HO-1 antibody (Enzo Life Sciences) was added to each sample, and the samples were incubated at room temperature for 30 minutes. Samples were washed with wash buffer and 1 µl of FITC goat anti-rabbit IgG (Sigma) was added as a secondary antibody. Samples were incubated at room temp for 30 minutes, washed with wash buffer and then resuspended in 600 µl 2% paraformaldehyde (Sigma). Monocytes, identified by PE-anti-CD14 staining, were analyzed for HO-1, expressed as the FITC GMFI after subtracting the GMFI for cells stained with the secondary antibody alone.

### Analysis of Control Monocyte Response to Plasma from Trauma Patients

The use of human peripheral blood monocytes from healthy donors was approved by the Human Research Review Committee of the University of New Mexico Health Sciences Center. Blood from healthy volunteers was drawn into heparinized tubes. Peripheral blood mononuclear cells (PBMC) were obtained by gradient separation using Ficoll Paque Plus (GE Healthcare). Briefly, 5 ml of whole blood was diluted 1∶1 with PBS and carefully layered over 3 ml of Ficoll Paque Plus. Samples were then centrifuged at 1400 rpm for 30 minutes and the mononuclear cell layer was collected. PBMC were washed two times in PBS and resuspended in RPMI-1640 medium (containing 10% heat-inactivated FBS-HyClone, Sigma). CD14+ monocytes were then positively selected to >90% purity using MACS magnetic bead separation (Miltenyi) according to manufacturer’s instructions. CD14+ cells were then resuspended at a concentration of 2×10^6^/ml and incubated with 25% trauma patient plasma or 25% healthy volunteer plasma for 44 hours at 37°C in a CO_2_ incubator. Patient plasma samples with high percentages of CD14^high^CD16^+^ monocytes were used in the trauma plasma incubation experiments. A dose response was performed with patient plasma on monocyte surface marker expression and a volume of 25% was the smallest concentration that remained effective, and was thus used for all the plasma incubation experiments (data not shown). Control samples were treated with volunteer plasma in a similar fashion. After the incubation period, samples were washed with PAB. CD16 and CD163 expression and intracellular HO-1 levels in the treated monocytes were determined as described above by flow cytometry. CD16 expression was expressed as a log transformation of the ΔGMFI. CD163 and HO-1 were expressed as GMFI. Intracellular TNF-α levels were determined following an additional 4 hour LPS stimulation (10 ng/ml) in the presence of Brefeldin A (10 µg/ml). TNF-α was expressed as the ΔGMFI. In separate experiments, plasma was depleted of CRP using p-aminophenyl phosphoryl choline agarose (Thermo Scientific) according to the manufacturer’s instructions and the expression of CD16, CD163 and intracellular HO-1 and TNF-α were determined as described above. Plasma CRP levels were determined before and after depletion by ELISA, and CRP was reduced to <1% of beginning levels following depletion. Similar experiments were done with the addition of the TGF-β receptor inhibitor, SB431542 (1 µmol, R&D Systems) and monocyte chemotactic protein 1 (MCP-1) monoclonal antibody (30 µg/ml, BD Bioscience) during the incubation with trauma patient plasma and control plasma [21]. We also evaluated the effects of M-CSF inhibition on CD16 expression with the M-CSF receptor inhibitor, Ki20227 (100 nm, Symansis) [22]. Because we found Ki20227 to be toxic to cells when incubated for longer than 24 hours, in these experiments, healthy volunteer monocytes were incubated with 25% trauma patient or control plasma with the M-CSF receptor inhibitor, Ki20227 or control solution, for 22 hours. The plates were then centrifuged and supernatants were decanted and replaced with 200 µl of RPMI-1640 medium. The monocytes were then incubated for another 22 hours and then washed and stained as described above to determine CD16 expression by flow cytometry. Cells treated with inhibitors showed no decreased viability by trypan blue staining.

### Control Monocyte Response to M-CSF Treatment

M-CSF has been shown to induce differentiation of monocytes to a CD16+ phenotype [23]. Positively selected CD14+ monocytes were isolated as described above and then resuspended at a concentration of 2×10^6^/ml and incubated with M-CSF (10 ng/ml) for 48 hours. M-CSF induced CD16 expression in normal healthy volunteer monocytes. Purified monocytes from healthy donors were incubated with 200 µg/ml CRP for 24 h and supernatants were analyzed for pro-inflammatory and anti-inflammatory cytokines. A portion of the same purified monocytes were incubated for 2 days in culture with M-CSF (10 ng/ml) followed by treatment with 200 µg/ml CRP for 24 h and similarly analyzed.

### Statistical Analysis

Graphical and statistical analyses were performed using Prism software version 5.0 (GraphPad, La Jolla, CA) and SAS (Statistical Analysis Software, Cary, NC). Spearman correlations were used to analyze patient data. Nonparametric Wilcoxon test was used to compare mean values of CD16, TNF-α, HO-1 and CD163 for monocytes incubated with plasma from trauma patients and healthy volunteers. Statistical significance was determined at *p*<0.05.

## Results

### CD14^high^CD16^+^ Monocytes in Trauma Patients

**Figure 1:**
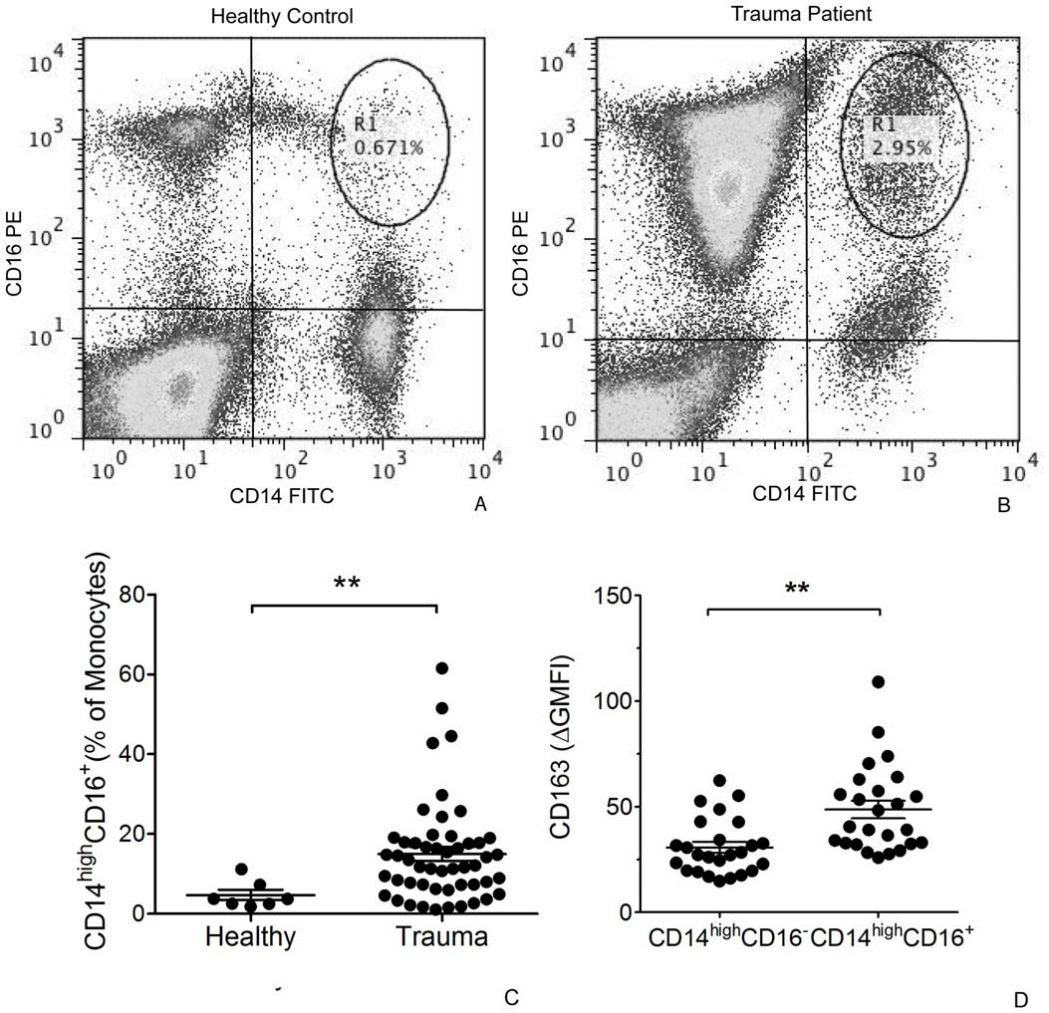
Representative flow cytometry results for CD14^high^CD16^+^ monocytes in healthy volunteer (A) and trauma patient (B) samples. Quadrants were established using appropriate isotype control antibodies. Increased numbers of the CD14^high^CD16^+^ monocytes subpopulation were seen in trauma patients (n = 50) compared to healthy volunteers (n = 7) (C). Surface CD163 expression was greater in CD14^high^CD16^+^ monocytes compared to CD14^+^CD16^−^ classic monocytes in 25 consecutive trauma patients (D). **, *P*<0.01, as determined by t-test.

**Figure 2:**
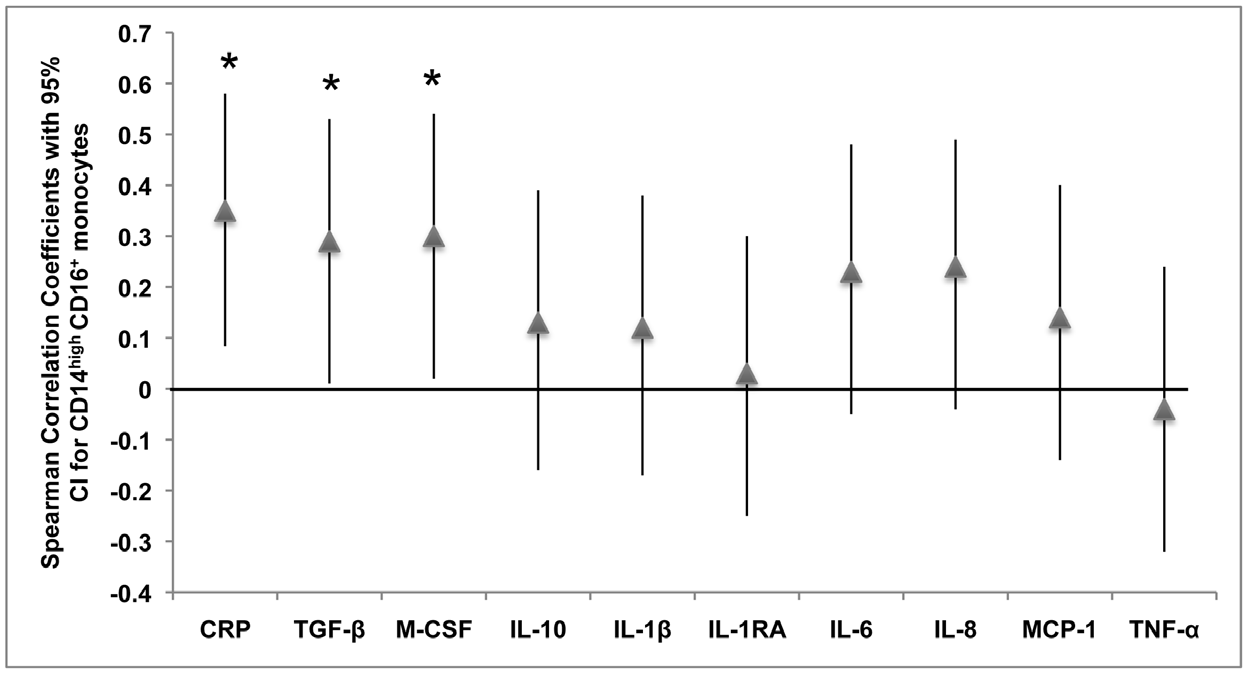
Spearman correlation coefficients with 95% confidence intervals for each factor as a predictor of the percentage of CD14^high^CD16^+^ monocytes (n = 50). *, *P*<0.05.

We evaluated monocyte surface markers in whole blood of trauma patients using flow cytometry. We identified increased numbers of the subpopulation of monocytes expressing high level of CD14 and CD16 in trauma patients (Figure 1). This cell population represented 11% or less of monocytes in healthy volunteers, but up to 60% of monocytes in trauma patients. Overall the mean percentage of CD14^high^CD16^+^ monocytes in patients vs. controls was 14.98±1.8 vs. 4.69±1.3, p<0.001. CD14^high^CD16^+^ monocytes expressed variable levels of HLA-DR, but overall HLA-DR expression was decreased from controls (data not shown). We found that the percentage of CD14^+^CD16^−^ “classical” monocytes were not significantly decreased in trauma patients compared to healthy controls (73.63±3.09 vs. 65.81±3.66, p>0.05) and the percentage of CD14^low^CD16^+^ was essentially unchanged in patients compared to controls (5.81±1.42 vs. 3.77±0.41, p>0.05). Since the hemoglobin scavenger receptor, CD163, has been described on the CD14^high^CD16+ monocyte subpopulation [11], [16], [17], we began evaluating CD163 expression on patient monocytes after noting this subpopulation increase in our patients. We found greater CD163 expression on CD14^high^CD16^+^ monocytes compared to CD14^high^CD16^−^ classical monocytes in the next 25 consecutive trauma patients we analyzed (Figure 1), consistent with alternative activation into regulatory CD14^high^CD16^+^ monocytes that have been described in other conditions [17], [24]. Plasma CRP levels, TGF-β levels and M-CSF levels, as determined by ELISA, highly correlated with the percentage of CD14^high^CD16^+^ monocytes in trauma patients (Figure 2).

### Control Monocyte Response to Plasma from Trauma Patients

**Figure 3:**
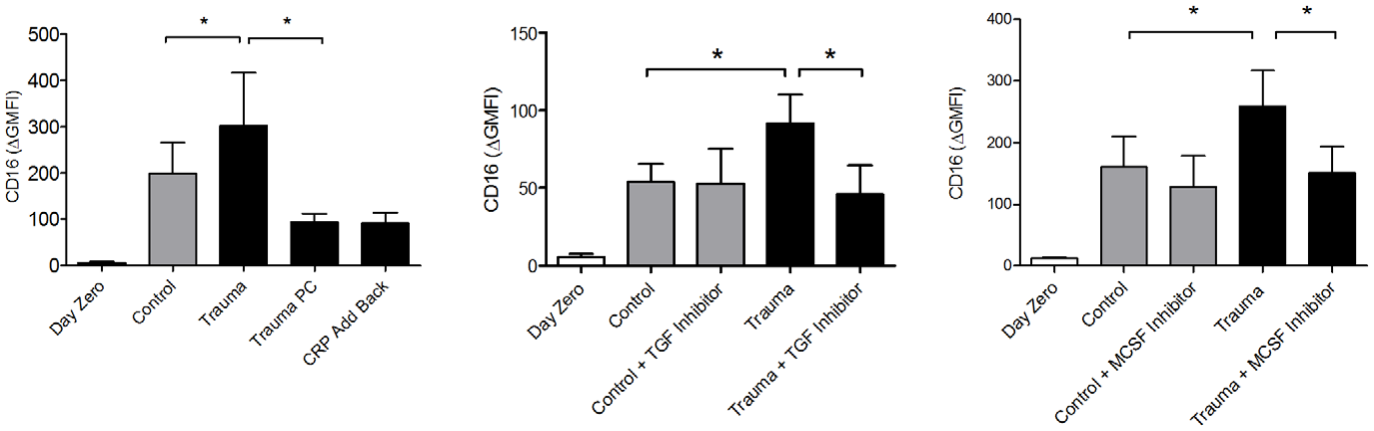
Surface CD16 expression on CD14^+^ monocytes before and after incubation with control or trauma patient plasma for 22 hours was decreased with CRP depletion (Trauma PC), but was not restored with CRP add-back experiments. TGF-β receptor inhibition with SB431542 (TGF Inhibitor) and M-CSF receptor inhibition with Ki20227 (M-CSF Inhibitor) significantly reduced CD16 expression of normal monocytes. * P<0.05; nonparametric Wilcoxon test was used to determine statistical significance; n = 6 for each separate experiment.

In order to determine the factors responsible for monocyte differentiation after trauma, we incubated monocytes from healthy volunteer donors with plasma from controls and from trauma patients. Incubation of healthy volunteer monocytes with plasma from trauma patients increased CD16 expression compared to control plasma. We then looked at the effects of CRP, TGF-β, M-CSF and MCP-1 on CD16 expression in this *in vitro* setting. We found that in donor monocytes incubated with trauma plasma, CD16 expression while decreased with CRP depletion, did not change with add-back experiments (Figure 3), suggesting CRP is not responsible for CD16 expression. We then looked at the effects of the TGF-β receptor inhibitor, SB431542, on CD16 expression after incubation of healthy monocytes with trauma plasma. We found that TGF-β receptor inhibition negated the increase in CD16 expression in donor monocytes incubated in trauma patient plasma (Figure 3). We found that M-CSF receptor inhibition with Ki20227 attenuated CD16 expression on control monocytes incubated with trauma patient plasma, similar to the findings seen with TGF-β receptor inhibition. We found that MCP-1 inhibition had no effect on CD16 expression on control monocytes incubated with trauma patient plasma (data not shown).

**Figure 4:**
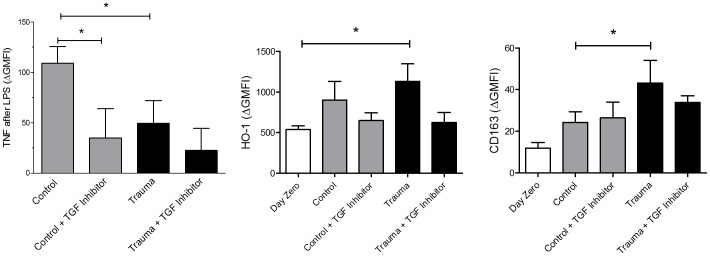
Intracellular HO-1 levels and surface CD163 expression were not affected by TGF-β receptor inhibition with SB431542 (TGF Inhibitor) on CD14^+^ monocytes incubated with control or trauma patient plasma for 22 hours. TNF-α response to LPS stimulation (10 ng/ml) was significantly diminished with SB431542. *, P<0.05; nonparametric Wilcoxon test was used to determine statistical significance; n = 6 for each separate experiment.

We then examined the effects of incubation of healthy volunteer donor monocytes with plasma from trauma patients on the expression of anti-inflammatory markers. Trauma plasma increased intracellular HO-1 expression compared to day 0 levels, but not over control plasma. Surface CD163 expression was increased after trauma plasma incubation compared to control plasma. At the same time, incubation of donor monocytes with trauma plasma caused a decrease in pro-inflammatory cytokine production. Trauma plasma attenuated the TNF-α response to LPS stimulation (Figure 4).

CRP depletion and add-back had no effect on HO-1 expression, CD163 expression or TNF-α production after LPS stimulation (data not shown). TGF-β receptor inhibition had no effect on HO-1 expression or CD163 expression, but appeared to block the TNF-α response to LPS (Figure 4).

### Plasma Cytokine Concentrations in Trauma Patients

CRP did not appear to be essential for trauma plasma effects on CD16, CD163 or HO-1 expression on monocytes *in vitro*. However, we found that CRP levels measured on day 2 correlated with the anti-inflammatory cytokines IL-10 and IL-1RA in trauma plasma. CRP also correlated with IL-6 and the chemokines, IL-8 and MCP-1 (Table 1). This suggested that CRP might be important for activating the differentiated monocyte population to produce cytokines.

**Table 1:** Spearman correlation coefficients with 95% confidence intervals for each factor with CRP measured at 48 h.

	SpearmanCorrelationCoefficient	95% Confidence Interval
IL-10	0.39	(0.17–0.61)**
IL-1RA	0.32	(0.03–0.56)*
IL-6	0.48	(0.23–0.68)**
IL-8	0.38	(0.10–0.60)**
MCP-1	0.37	(0.09–0.59)**
TGF-β	0.08	(−0.22–0.37)
M-CSF	0.28	(−0.02–0.53)
TNF-α	0.19	(−0.10–0.46)
IL-1β	0.03	(−0.26–0.32)
HO-1	0.15	(−0.14–0.42)

CRP correlates significantly with the anti-inflammatory cytokines IL-10 and IL-1RA, as well as IL-6 and the chemokines, IL-8 and MCP-1.

* P<0.05,

** P<0.01, n = 50. IFN-γ and IL-12 levels are not included because they were not present in significant amounts to account for in final analysis.

### Control Monocyte Response to CRP

**Figure 5:**
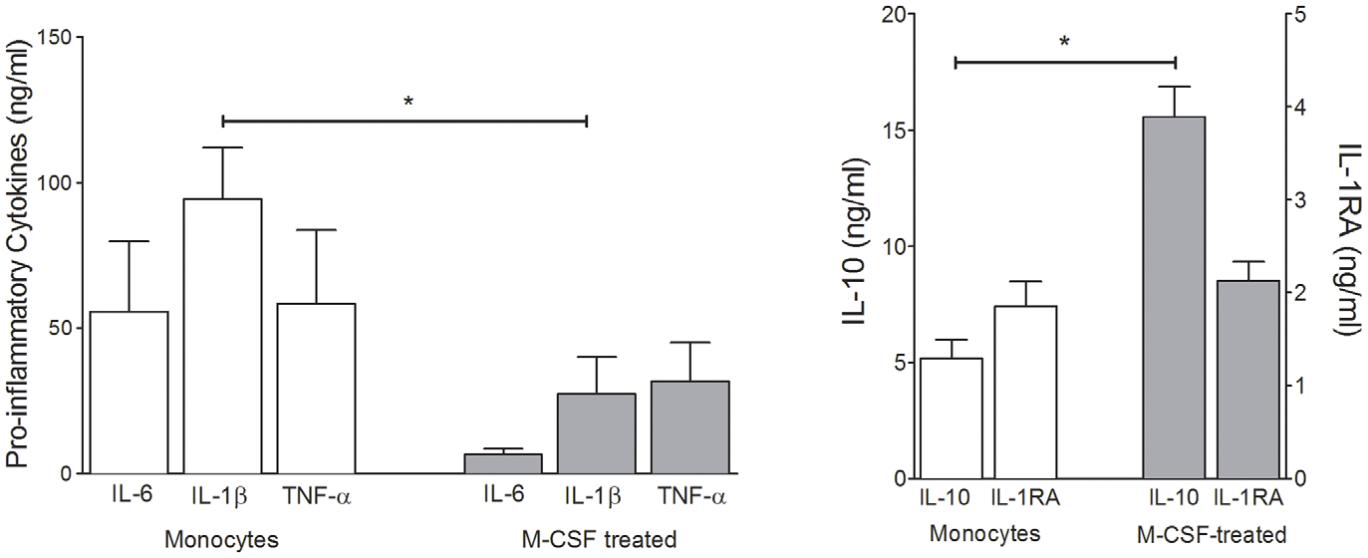
Purified monocytes and M-CSF treated (10 ng/ml) purified monocytes from the same donor were incubated with CRP (200 µg/ml) for 24 h and supernatants were analyzed for pro-inflammatory and anti-inflammatory cytokines. Control values were several thousand-fold less than treated values. Monocytes secreted pro-inflammatory cytokines in response to CRP, which decreased after M-CSF differentiation. The differentiated monocytes secreted anti-inflammatory cytokines, IL-1RA and IL-10. *, P<0.05 vs. purified monocytes; nonparametric Mann Whitney test was used to analyze differences between groups. Mean ±SEM of triplicate wells from 3 independent experiments.

We then evaluated the effects of acute phase levels of CRP on monocytes from healthy volunteers compared to the same monocytes after M-CSF differentiation to CD14^high^CD16^+^ monocytes *in vitro*. Healthy donor monocytes treated with CRP secreted the pro-inflammatory cytokines TNF-α, IL-6 and IL-1β with low levels of anti-inflammatory cytokines, IL-1RA and IL-10. In contrast, M-CSF differentiated monocytes secreted lower amounts of pro-inflammatory cytokines and greatly increased IL-10 (Figure 5). Although we recognize that many cell types contribute to the plasma levels of cytokines, these results are consistent with the correlation between plasma CRP levels and plasma cytokines in trauma patients.

### Patient Outcomes

Of the 50 severely injured patients enrolled in this study, 26 developed infectious complications and 6 developed multiple organ failure. However, none of these outcomes correlated with the expansion of CD14^high^CD16^+^ monocyte subpopulation in patients.

## Discussion

This paper represents the first description of the CD14^high^CD16^+^ monocyte subpopulation in trauma patients. We found a clear expansion of the CD14^high^CD16^+^ subpopulation of monocytes in severely injured trauma patients that correlated strongly with plasma concentrations of TGF-β, M-CSF and the acute phase protein CRP. We demonstrated generation of cells with this phenotype *in vitro* using trauma plasma or M-CSF. Differentiation of monocytes by trauma plasma was blocked by a TGF-β receptor inhibitor as well as an M-CSF receptor inhibitor. Monocytes differentiated with M-CSF were activated by acute phase levels of CRP to produce anti-inflammatory cytokines. These findings support those of Skrzeczyriska-Moncznik *et al.* in which the CD14^high^CD16^+^ monocyte subpopulation was found to have anti-inflammatory properties [11]. We also showed an expression of the CD163 hemoglobin scavenger receptor, an indicator of alternative activation, on this subpopulation of monocytes in trauma patients. Others have shown that stimulation of the CD163 receptor with hemoglobin-haptoglobin complexes induce IL-10 secretion and HO-1 synthesis [11], [16], [17] in these monocytes.

In contrast to our findings, Kampalath *et al.* showed a significant increase in CD16^−^ monocyte subsets in post-trauma patients compared to controls [25]. However, in their paper, the severity of injury of the post-trauma patients is not defined. Additionally, the timing of blood draw is unclear and is only defined as during the first visit to the emergency department.

CD14^high^CD16^+^ monocytes have been shown to expand in various inflammatory conditions, including sepsis, asthma, breast cancer and chronic hepatitis B (HBV) infection [26], [27], [28]. There is some debate on whether these monocytes are considered pro-inflammatory or anti-inflammatory since in the majority of clinical reports, CD16+ monocytes have been analyzed and catalogued as one single subset. In a recent study of breast cancer patients, Feng *et al* did not recognize two distinct subpopulations of CD16^+^ monocytes and described them as pro-inflammatory [26]. In HBV infected patients, while the CD14^high^CD16^+^ monocytes are described as a distinct group, the investigators did not report anti-inflammatory cytokine secretion from the two populations [28]. Skrzeczyriska-Moncznik *et al.* demonstrated that CD14^high^CD16^+^ monocytes produced comparable amounts of the pro-inflammatory cytokine TNF-α with different stimuli, while maintaining high IL-10 production, suggesting an anti-inflammatory proclivity [11].

This study was limited to examining the monocyte populations early following trauma. It is possible that an effect of regulatory monocytes on monocyte deactivation would become apparent later in the course of the compensatory anti-inflammatory response. It is also possible that the CD14^high^CD16^+^ monocyte subpopulation represents alternatively activated monocytes/macrophages with “wound-healing” rather than regulatory activity, as these also express CD16 and CD163 [29], [30].

TGF-β has been shown to induce CD16 expression on PBMC *in vitro* [31], and our findings support that TGF-β in trauma patient plasma is responsible for increasing CD16 expression on CD14+ cells. We demonstrated that inhibition of the TGF-β receptor negated CD16 expression after exposure to trauma patient plasma. M-CSF has been shown to induce monocyte differentiation from the classic monocytes population to a macrophage-like CD16^+^ subpopulation [23], [32], and we found that M-CSF levels in trauma patients were elevated compared to normal healthy volunteers. Additionally we found that inhibition of the M-CSF receptor attenuated CD16 expression, although to a lesser extent than inhibition of the TGF-β receptor, in donor monocytes after exposure to trauma patient plasma. Feng *et al* demonstrated that purified CD14^+^ monocytes exposed to MCP-1 increased CD16^+^ expression and neutralizing antibodies against MCP-1 inhibited this expansion [26]. We also looked at inhibition of the chemokine MCP-1 on CD16 expression. We found that inhibition of MCP-1 failed to affect CD16 expression in normal healthy volunteer monocytes incubated with trauma patient plasma.

We also demonstrated that inhibition of the TGF-β receptor impaired TNF-α expression, confirming the findings of Chen *et al* [33]. In their study, they demonstrated that blockade of the receptor targeted by SB431542, activin receptor-like kinase 5 (ALK5), reduced LPS-induced TNF-α protein levels, while TNF-α mRNA remained the same. They suggested that TGF-β signaling plays a general role in LPS-induced TNF-α expression [33].

CRP is an acute phase reactant. Its plasma concentration increases from <5 to >100 µg/ml within 48 h of injury due to increased synthesis by hepatocytes responding to IL-6. Because acute phase levels of CRP have been shown to induce M-CSF release via upregulation of NF-κB [34] we evaluated the effects of CRP on monocyte expression of CD16, CD163 and HO-1. Highly elevated CRP levels are seen in trauma, and maintenance of these levels is associated with a poor prognosis [35]. We demonstrated that CRP levels following traumatic injury correlate with the expansion of the CD14^high^CD16^+^ monocyte subpopulation, but acute phase levels of CRP were not responsible for CD16 monocyte differentiation by trauma plasma *in vitro* as determined by depletion and add-back experiments. We were unable to confirm induction of M-CSF synthesis by CRP treated PBMC (data not shown). CD16 is FcγRIII, one of the receptors for CRP on myeloid cells [36]. Therefore, we also examined the effect of CRP on activation of CD16-expressing monocytes. Exposure of M-CSF differentiated monocytes *in vitro* to acute phase levels of CRP increased the secretion of anti-inflammatory cytokines. This suggests that CD14^high^CD16^+^ monocytes may represent a regulatory monocyte population in trauma patients, and that CRP may act on these cells to increase synthesis of anti-inflammatory cytokines. Activation of mouse regulatory macrophages by co-stimulation of FcγR and TLR has been described for both IgG and CRP [37], [38].

Overall our study has demonstrated expansion of a distinct monocyte subpopulation with markers of alternatively differentiated monocytes/macrophages in addition to the previously described deactivated monocytes following trauma. These cells are CD14^high^CD16^+^, similar to the population described as “intermediate” monocytes [8]. However, they also express a marker of alternative monocyte/macrophage activation and differentiation, CD163 [15]. Our results implicate TGF-β and M-CSF in the differentiation of this subpopulation, and CRP as a potential activator of these cells for cytokine production. Additional studies are underway to determine the functional role of these monocytes in immune regulation following trauma.
